# Sensitivity Enhancement of Silicon-on-Insulator CMOS MEMS Thermal Hot-Film Flow Sensors by Minimizing Membrane Conductive Heat Losses

**DOI:** 10.3390/s19081860

**Published:** 2019-04-18

**Authors:** Zahid Mehmood, Ibraheem Haneef, Syed Zeeshan Ali, Florin Udrea

**Affiliations:** 1 Engineering Department, University of Cambridge, Cambridge CB3 0FA, UK; fu10000@hermes.cam.ac.uk; 2 Institute of Avionics & Aeronautics, Air University, E-9, Islamabad 44000, Pakistan; ibraheem.haneef@mail.au.edu.pk; 3 AMS Sensors UK Ltd., Deanland House, 160 Cowley Road, Cambridge CB4 0DL, UK; Zeeshan.Ali@ams.com

**Keywords:** MEMS thermal flow sensors, review, conduction losses, heater/hot-film, membrane shape, membrane to heater ratio, silicon-on-insulator (SOI), complementary metal oxide semiconductor (CMOS)

## Abstract

Minimizing conductive heat losses in Micro-Electro-Mechanical-Systems (MEMS) thermal (hot-film) flow sensors is the key to minimize the sensors’ power consumption and maximize their sensitivity. Through a comprehensive review of literature on MEMS thermal (calorimetric, time of flight, hot-film/hot-film) flow sensors published during the last two decades, we establish that for curtailing conductive heat losses in the sensors, researchers have either used low thermal conductivity substrate materials or, as a more effective solution, created low thermal conductivity membranes under the heaters/hot-films. However, no systematic experimental study exists that investigates the effect of membrane shape, membrane size, heater/hot-film length and Membrane (size) to Heater (hot-film length) *Ratio* (*MHR*) on sensors’ conductive heat losses. Therefore, in this paper we have provided experimental evidence of dependence of conductive heat losses in membrane based MEMS hot-film flow sensors on *MHR* by using eight MEMS hot-film flow sensors, fabricated in a 1 µm silicon-on-insulator (SOI) CMOS foundry, that are thermally isolated by square and circular membranes. Experimental results demonstrate that: (a) thermal resistance of both square and circular membrane hot-film sensors increases with increasing *MHR*, and (b) conduction losses in square membrane based hot-film flow sensors are lower than the sensors having circular membrane. The difference (or gain) in thermal resistance of square membrane hot-film flow sensors viz-a-viz the sensors on circular membrane, however, decreases with increasing *MHR*. At *MHR* = 2, this difference is 5.2%, which reduces to 3.0% and 2.6% at *MHR* = 3 and *MHR* = 4, respectively. The study establishes that for membrane based SOI CMOS MEMS hot-film sensors, the optimum *MHR* is 3.35 for square membranes and 3.30 for circular membranes, beyond which the gain in sensors’ thermal efficiency (thermal resistance) is not economical due to the associated sharp increase in the sensors’ (membrane) size, which makes sensors more expensive as well as fragile. This paper hence, provides a key guideline to MEMS researchers for designing the square and circular membranes-supported micro-machined thermal (hot-film) flow sensors that are thermally most-efficient, mechanically robust and economically viable.

## 1. Introduction

Flow sensors are extensively used for flow measurements in diverse applications in different fields including aerospace [1,2,3,4,5,6], automotive [7], biomedical [8,9,10,11], environmental [12,13,14,15,16,17], hydrodynamics [18] and the chemical and process industries [19]. They can be broadly classified as either non-thermal or thermal [20].

Non-thermal flow sensors can be grouped, as per their transduction scheme, into differential pressure-based, lift force-based, cantilever deflection-based and resonating frequency-based. A comprehensive review of these non-thermal flow sensors is presented by Wang et al. [21]. Thermal flow sensors, on the other hand, use temperature as the main measurand parameter. State of the art reviews of these sensors can be found in [20,21]. These have been investigated extensively because of their structural simplicity (no moving parts) and robustness, easy electrical interface, high temporal and spatial resolution, low cost and high reliability when mass fabricated using silicon technology [22,23]. According to their measurement techniques, thermal flow sensors can be segregated into three main types (or configurations): (a) calorimetric flow sensors [3,5,6,15,17,24,25,26,27,28,29,30,31,32,33,34,35,36,37,38,39,40,41,42,43,44,45,46,47,48,49,50,51,52,53,54,55,56,57,58], (b) time of flight (TOF) flow sensors [36,59,60,61,62] and (c) hot-wire (HW)/hot-film(HF)flow sensors [4,5,8,9,10,11,13,18,22,23,27,34,36,42,50,63,64,65,66,67,68,69,70,71,72,73,74,75,76,77,78,79,80,81,82,83,84,85,86,87,88,89,90,91,92,93,94,95,96,97,98,99,100,101,102,103,104,105,106,107,108,109,110,111,112,113,114,115].

Calorimetric thermal flow sensors are typically used to measure flow velocity [45,46,48,49], flow direction [17,47,48,49] and flow rate [51,52], whereas TOF thermal flow sensors are generally used to extract only flow velocity and flow rate [60,62]. Hot-film [11,71,77,110,111,112,114] and hot-wire [116] based thermal flow sensors have been used to measure flow velocity, mass/volume flow rate and fluidic wall shear stress.

Although calorimetric flow sensors have the highest sensitivity among the three types of thermal flow sensors, they however require at least two additional temperature sensors for extracting flow information [20,44]. In TOF thermal flow sensors, a thermal pulse, injected into a flow at point A, is detected downstream at point B. The total time taken by the pulse is then used for the flow measurements. This technique, however, has many drawbacks; i.e., requirement of injecting a thermal peak with enough energy to be detected downstream, rapid decay of peak due to longitudinal heat diffusion, conduction through the walls and Taylor dispersion [62]. Among these three measurement techniques, owing to its simple fabrication and implementation process, short response time and large flow measurement range, the hot-wire/hot- film configuration is the most widely investigated and adopted approach [58].

In the hot-wire/hot-film thermal flow sensors, a thin wire/film (often referred as hot-wire/hot-film or heater), placed over a low-thermal conductivity substrate or membrane, is heated above the ambient temperature. The amount of heat convected to the fluid passing over the hot-film/hot-wire is then transduced in terms of varying voltage of the heater to extract the flow information [20]. Since the fluid passing over the sensor only takes away heat through convective heat transfer, therefore, it is desirable to increase the convective heat transfer and reduce the conductive heat transfer [65] to the substrate.

Sensitivity and power consumption of all three types of thermal flow sensors (i.e., calorimetric, TOF, HW/HF) depends upon their ability to maximize convective heat transfer to the flow and minimize conduction losses to the substrate. To achieve this objective, researchers have resorted to two strategies: (a) use of low thermal conductivity substrate materials, and (b) thermal isolation of hot element by creating membranes under the heater/hot element.

In all three types of MEMS thermal flow sensors, the materials used as a substrate for minimizing conductive heat loss (from the heater/sensor to the substrate) are either ceramics or polymers. A comprehensive overview of ceramics and polymers used as substrate under different MEMS thermal flow sensors (heaters) is given in Table 1 and Table 2, respectively.

The ceramic materials with low thermal conductivity (‘k’), used as substrate in MEMS thermal flow sensors and summarized in Table 1, includeglass (k = 1.1 W/(m-K) [57]), porous silicon (k = 1.2 W/(m-K) [45]), silicon nitride (k = 20 W/(m-K) [118]), aluminum oxide (k = 20 W/(m-K) [49], as-grown polysilicon (k = 13.8 W/(m-K) and amorphous recrystallized polysilicon (k = 22 W/(m-K) [119]). Silicon, which has relatively higher thermal conductivity (k  = 130 W/(m-K) [51]) has also been used as a substrate material in few cases. However, such substrates have generally been used in wind sensing applications, where sensors are mostly wafer thick [15].

Thermal conductivity of polymers is typically lower than the ceramics and these are also good candidate substrate materials, as summarized in Table 2, for reduced conduction losses in MEMS thermal flow sensors. Polyimide, with a thermal conductivity of k = 0.29 W/(m-K), has been most widely used as a substrate material [11,52,58,96,97,98,100,106,111,115]. Other substrate materials from polymers family include SU-8 (k = 0.2 W/(m-K) [50], PMMA (k = 0.1920–0.2 W/(m-K), pyrex (k = 1.3–1.5 W/(m-K) [120], Kapton (k = 0.12 W/(m-K) [121]), parylene N (k = 0.13 W/(m-K) [84]) and parylene C (k = 0.082 W/(m-K) [122]. Although, parylene N has higher thermal conductivity than parylene C, yet due to its higher melting temperature (420 °C), it is preferred as membrane material for the hot-film sensors over parylene C, which has a melting temperature of 290 °C [74].

Similarly, a review of the membrane-mounted MEMS thermal flow sensors illustrates that the membranes used for thermal isolation of the sensing heater (hot-element) are made of diverse materials and have different shapes and sizes. Most of these membranes are made of ceramics, with a handful made of polymer materials. For a quick reference, the MEMS thermal flow sensors having membranes of square, circular and rectangular shapes are grouped and their key information is summarized in Table 3, Table 4 and Table 5, respectively.

A quick look at Table 3, Table 4 and Table 5 reveals that among ceramics, silicon nitride is the most widely used membrane material for thermal isolation of MEMS thermal flow sensors [22,25,26,33,39,59,60,63,64,65,67,68,69,70,71,72,73,75,76,77,78,79,80,81,85,86,89,127]. To further reduce the heat conduction, bi-layer membranes of silicon nitride and silicon oxides have also been reported [3,8,24,30,31,34,42,83,117]. Thermal conductivity of this bi-layer system depends upon the individual thickness of silicon nitride and silicon oxide layers. However, a typical thermal conductivity value for this bi-layer system is k = 1.98 W/(m-K) [3].

Because of much lower thermal conductivity values, polyimide [40,41,92,94], parylene C [23,82] and parylene N [74,84] are the three commonly used membrane materials in thermal flow sensors from the polymers family.

Like other devices with membranes [128,129], besides choice of suitable materials, membrane shape and membrane [side length (for square membrane) or diameter (for circular membrane)] to heater (hot-film (length) ratio (MHR) are two other design variables that are required to be optimized in thermal flow sensors employing membranes for minimization of conduction heat losses from the heater (hot-film) to the substrate.

As mentioned earlier, membranes of three shapes (i.e., square, circular and rectangular) with different sizes (Table 3, Table 4 and Table 5) have been used in different MEMS thermal flow sensors reported in the literature. An analysis of these membranes-based hot-film thermal flow sensors reveals that the MHR of these sensors fall in the range of 1.0–14.3 (Table 3—square membranes), 1.0–3.0 (Table 4—circular membranes) and 1.25–1.66 (Table 5—rectangular membranes), respectively. Interestingly, it is only tacitly assumed in all the reports listed in these three tables that the conductive heat losses depend largely on MHR, since none of the studies published during the last two decades (Table 3, Table 4 and Table 5) has explored the effect of MHR on the thermal efficiency (thermal resistance) of the sensors and the conductive heat losses from the sensor (hot-film/heater) to the substrate through formal experiments.

In this paper, therefore, a systematic experimental investigations of the effects of MHR and membrane shape (square versus circular) on the thermal resistance (or conductive heat losses) of MEMS thermal [thin (hot) film] flow sensors are reported for the first time.

The scope of the paper is limited to minimize the conductive heat losses to the substrate, thereby achieving improved thermal performance of the hot-film flow sensors. The heat losses due to radiation and natural or forced convection have not been taken into account in this study.

The experimental investigation is carried on MEMS thermal (hot-film) flow sensors having silicon oxide membranes (with a very thin silicon nitride passivation layer on the top to protect the sensors and power tracks), fabricated through a 1 µm Silicon-on-Insulator (SOI) Complementary Metal Oxide Semiconductor (CMOS) process. It is pertinent to highlight that silicon oxide has very low thermal conductivity (k = 1.4W/(m-K) [118]) and there are hardly any studies, excluding a few reported by our group [5,6,83,125,126,130,131], that utilize silicon oxide membranes for thermal isolation of MEMS thermal hot-film flow sensors produced through a commercial SOI CMOS process.

Eight such sensors, with two different membrane shapes (i.e., square and circular) and four Membranes to Heater length Ratios (i.e., MHR = 1, 2, 3 and 4) are fabricated and characterized. The membrane shape that achieves minimum conduction losses has been identified experimentally. The optimum membrane to heater ratios (MHR) for both square and circular membranes, beyond which further gain in thermal resistance of the sensor versus corresponding increase in sensor (membrane) size and related cost per sensor becomes un-economical, is also investigated.

The remaining paper is organized as follows: the design of SOI CMOS MEMS hot film sensor chips is presented in Section 2, followed by their fabrication in Section 3. Experimental results are discussed in Section 4. Finally, the conclusions are given in Section 5.

## 2. SOI CMOS MEMS Hot-Film Sensors Chip Design

**Figure 1:**
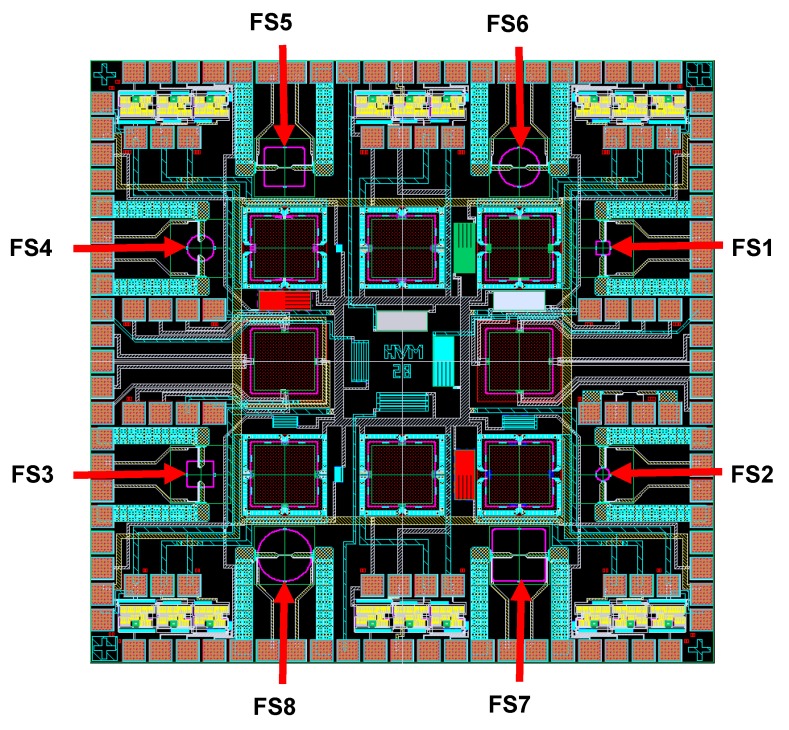
Cadence layout of the MEMS sensor chip which contains eight hot-film thermal flow sensors (FS1 to FS8) in it. The hot-film length is 80 µm for all eight sensors. However, membrane side length/diameter for FS1 & FS2, FS3 & FS4, FS5 & FS6 and FS7 &FS8 is 80, 160, 240 and 320 µm, respectively. FS1, FS3, FS5 and FS7 (odd numbers) have square membranes, while FS2, FS4, FS6 and FS8 (even numbers) have circular membranes.

MEMS hot-film sensors were designed using Cadence ^TM^ Virtuoso^®^ layout editor. Figure 1 [131] is the Cadence layout of designed SOI CMOS MEMS multi sensor chip showing the layout of all eight sensors on the die. Eight flow sensors (i.e., FS1 to FS8), four with square and four with circular membranes, with each type having a MHR of 1, 2, 3 and 4, were designed. The dimensions (length × width × thickness) of the hot-film (i.e., 80 µm× 2 µm× 0.3 µm) were kept identical for all eight sensors. The square membranes’ side lengths and circular membranes’ diameters were maintained as 80 µm, 160 µm, 240 µm and 320 µm for achieving a MHR = 1, 2, 3 and 4, respectively.

**Figure 2:**
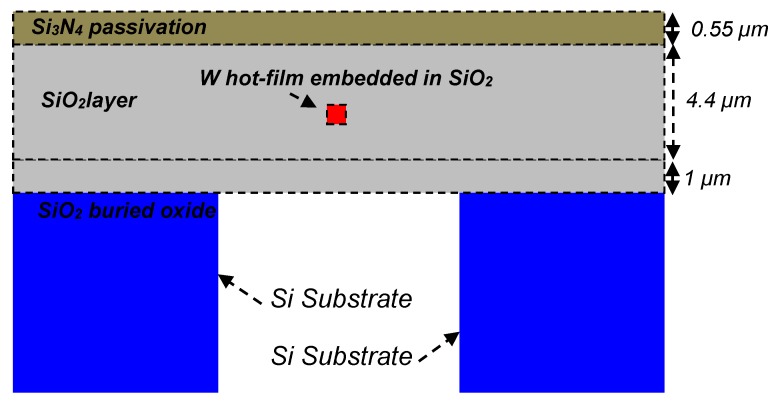
Schematic cross-section (not to the scale) of a tungsten hot-film thermal flow sensor.

The details of the geometries of all eight SOI CMOS MEMS thermal hot-film flow sensors are given in Table 6. Tungsten, because of its superior mechanical (tensile strength, Young’s modulus and density) and thermal properties (thermal conductivity and melting temperature) [6] is used as a hot-film and interconnects material. To achieve better thermal isolation and mechanical support for the hot-film, a 5.4 µm thick silicon dioxide membrane is used to embed/support the tungsten hot-film sensors. A 0.55 µm thick silicon nitride passivation layer is also deposited at the top of wafer to protect the metal tracks and sensors. The schematic cross-section of the tungsten hot-film designed thermal flow sensor is shown in Figure 2.

## 3. SOI CMOS MEMS Hot-Film Sensors Chip Fabrication

Hot-film sensors are fabricated using 1-µm SOI CMOS process in a commercial CMOS MEMS foundry followed by a post-CMOS Deep Reactive Ion Etching (DRIE) process for creating cavities under membrane to further increase the thermal isolation of the hot-film sensors. A silicon substrate embedded with a buried oxide layer (i.e., SOI wafer) is used. The SOI technology provides four basic advantages; i.e.,(a) the buried oxide acts as an etch stop layer for DRIE process, thus effectively controlling the etch depth and provides a uniform thickness to all sensor membranes, (b) provides a thermal isolation of sensing area, thus reducing the power losses to silicon substrate, (c) electrically isolates the electronic circuitry, reducing cross talk, and (d) increases the device operating temperatures range [132,133]. Cavities with vertical side walls are achieved using DRIE as it does not depend upon the lattice orientation of silicon substrate [6].

**Figure 3:**
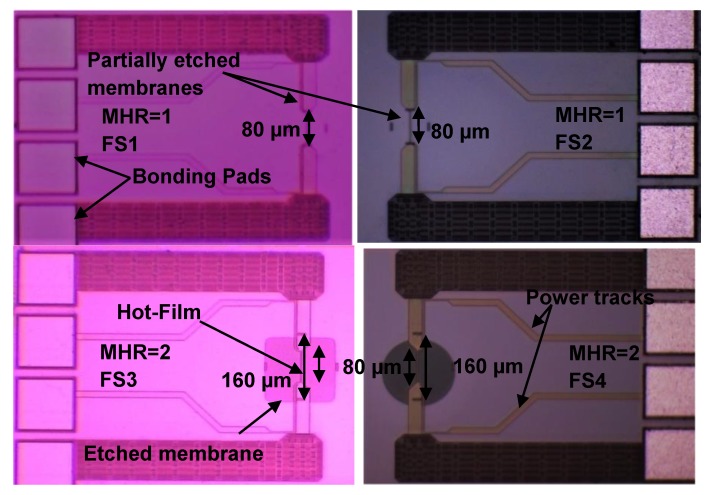
Optical micrographs (top view) of fabricated hot-film flow sensors having membrane to heater ratio MHR=1 (FS1 and FS2) and MHR=2 (FS3 and FS4).

**Figure 4:**
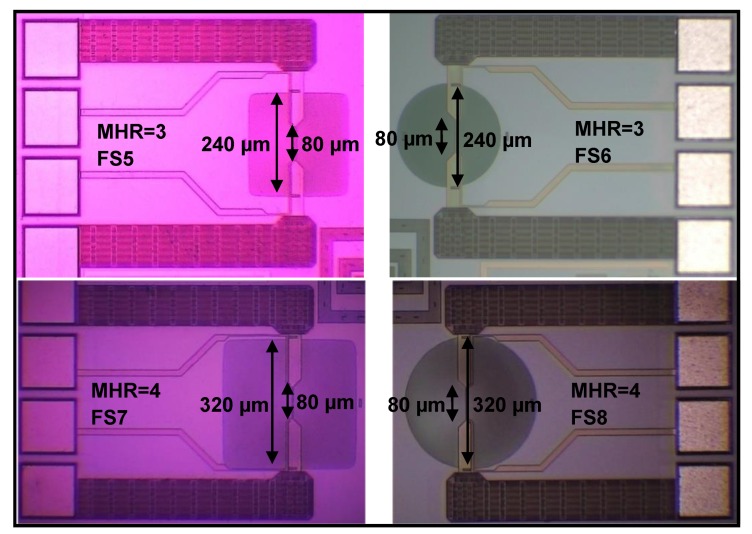
Optical micrographs (top view) of fabricated hot-film flow sensors having membrane to heater ratio MHR=3 (FS5 and FS6).

Optical micrographs of the fabricated sensors are shown in Figure 3 and Figure 4. It is interesting to note from the optical micrographs of FS1 and FS2 sensors (Figure 3) that the cavities are not created underneath these sensors during the post-CMOS DRIE processing. 

The micro-sensors chip (Figure 1) containing FS1 and FS2 sensors has a variety of other (i.e., pressure and temperature) sensors as well with the membrane dimensions ranging from 80 µm–400 µm. Due to micro-loading effects and aspect ratio dependent etching [134,135], the membranes with the larger opening got etched earlier and the membranes (of smallest sensor, FS1 and FS2) with smaller opening (i.e., diameter = 80 µm) remained un-etched or partially etched. Therefore, these sensors are actually fabricated on the full substrate or only partially etched substrate, and will be discussed accordingly.

## 4. Experimental Results and Discussion

**Figure 5:**
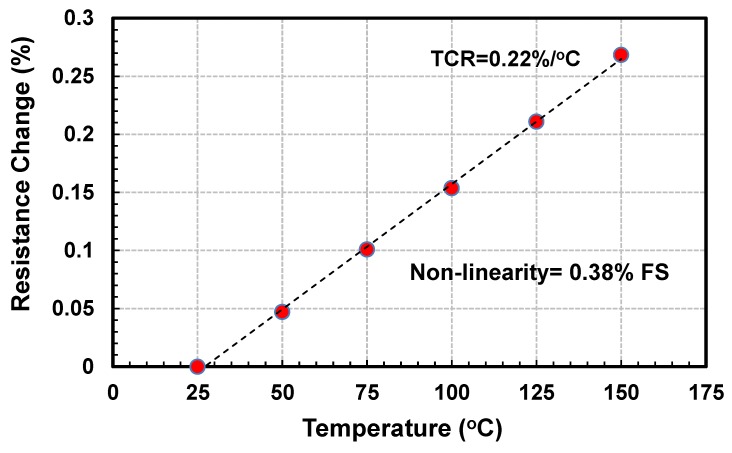
Temperature coefficient of resistance (TCR) of the tungsten based MEMS hot-film flow sensor.

A Labview (National Instruments, Austin, TX, USA) data acquisition system integrated with a Keithley 2400 Source and Measuring Unit (SMU, Tektronix, Inc., Beaverton, OR, USA) and a Model S-1160 probe station (Signatone Corp, Gilroy, CA, USA) equipped with temperature controller and hot chuck are used for sensors’ experimental (electro-thermal) characterization. In order to characterize the sensor for temperature coefficient of resistance (TCR), resistance variation of the tungsten heater from 25–150 °C is obtained. Figure 5 is the plot of the temperature versus percentage change in resistance of the hot-film heater. The slope of this curve is the TCR of the tungsten hot-film sensors, which is approximately 0.22%/°C. The TCR of the sensor is quite linear with a non-linearity of only 0.38% FS. A similar TCR value (0.21%/°C) has been reported earlier for tungsten thin film [83]. This TCR is almost double the value of polysilicon thin films (i.e., TCR of 0.13%/°C) [69] and an order of magnitude higher than the carbon nano tubes (i.e., TCR of 0.04 %/°C) [99].

**Figure 6:**
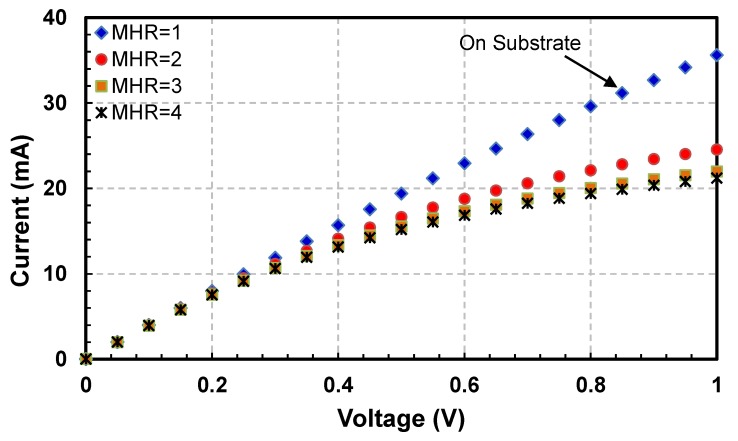
I−V curves of SOI CMOS MEMS hot-film sensors FS1, FS3, FS5 and FS7 having square membranes: For FS1 sensor with MHR=1 (that has partially etched membrane), the I-V curve is almost linear indicating negligible change in sensor’s resistance (i.e., no heating). With increasing MHR in FS3, FS5 and FS7 sensors (i.e., MHR=2, 3, 4, respectively), the Joule heating and resistance increase significantly.

**Figure 7:**
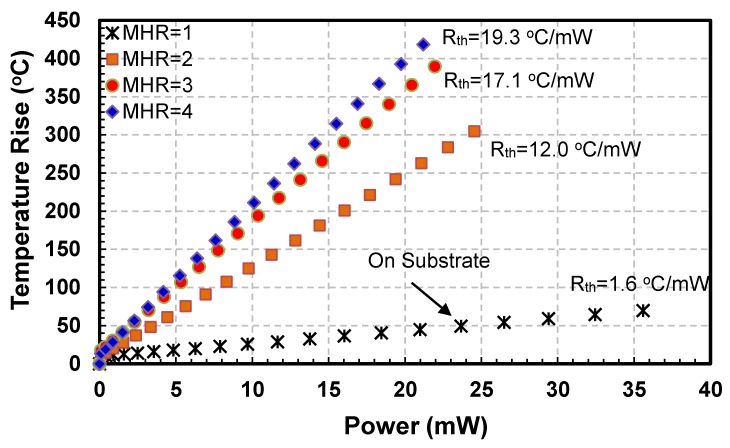
Power versus temperature rise curves for hot-film sensors FS1, FS3, FS5 and FS7 having square membranes: For FS1 sensor with MHR=1 (having partially etched membrane), there is a negligible rise in sensor temperature. For FS3, FS5 and FS7 sensors with higher MHRs (i.e., MHR=2, 3, 4, respectively), the temperature rise is significant, but its % increment between MHR=2 to MHR=3 and then from MHR=3 to MHR=4 reduces drastically.

Current-Voltage (I−V) and Power-Temperature (P−T) curves for all sensors are plotted to evaluate their electro-thermal characteristics. I−V curves for the four hot-film sensors having square membrane (i.e., FS1, FS3, FS5 and FS7 with MHR = 1, 2, 3 and 4, respectively) are shown in Figure 6 while their P−T curves are depicted in Figure 7.

The variations in I−V curves are different with changing MHR. For MHR=1 (FS1 sensor in Figure 3, which are actually fabricated on a partially etched substrate), current is almost directly proportional to the voltage (Figure 6). As expected, there is negligible joule heating and almost all the heat generated is being conducted straight into the substrate. The thermal resistance of the FS1 hot-film sensor is only 1.6 °C/mW (Figure 7). The effect of having a membrane under the sensor (i.e., thermal isolation of the hot-film sensor) is not visible in this plot as the membrane for FS1 was not created fully and only partial etching took place during the post-CMOSDRIE processing due to micro-loading effects and aspect ratio dependent etching [134,135].

**Figure 8:**
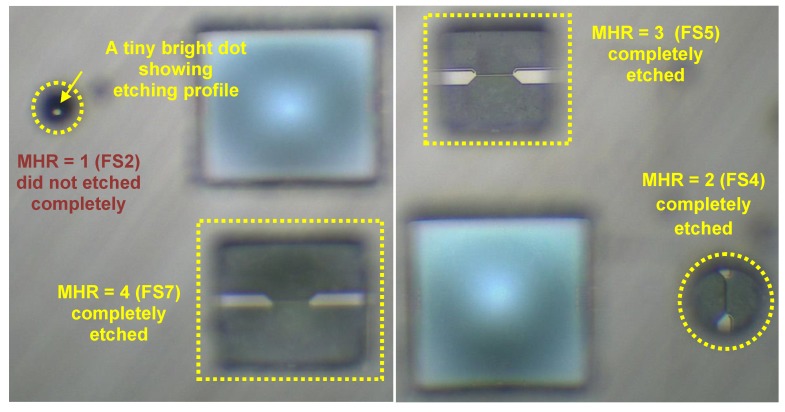
Optical micrograph of backside of the sensor chip, indicating the extent of etching for sensors with different MHRs. Hot-films are visible from the backside of the chip for MHR=2, 3 and 4, indicating a complete etching. For MHR=1, a tiny bright dot can be seen, indicating only a partial etching.

The backside picture of the MEMS sensors chip is shown in Figure 8. The hot-films of the completely etched sensors can be seen from the rear side of the sensor chip. However, for sensor having MHR =1, only a tiny bright dot can be seen, indicating a partial etching of the sensor. Thermal performance of un-etched thermal flow sensors has also been evaluated in the past. For example, Liang et al. [78] achieved a thermal resistance of 0.2 °C/mW for a titanium/platinum alloy strip of size 100 µm × 2 µm × 0.2 µm, directly fabricated on a silicon substrate. The thermal resistance in case of our FS1 sensor is comparatively higher, most likely, due to the fact that instead of silicon substrate a SOI substrate has been used, which has a very low thermal conductivity silicon oxide layer just underneath the hot-film. The other reason is that although all the silicon was not etched during the etching, still a partial etching did take place under the hot-film sensor (Figure 8), which reduced the amount of heat being lost to the substrate to some extent.

For FS3 sensor having square membrane with MHR=2 (Figure 3), I−V curve is nonlinear having decreasing slopes with increasing voltages (Figure 6). In this configuration, the amount of required current to the hot-film sensor is decreasing with the increasing voltage, which implies an increase in sensor’s temperature due to joule heating. As shown in the P−T curve (Figure 7), thermal resistance of the FS3 square membrane hot-film sensor with (MHR=2) is 12 °C/mW. FS3 sensor has an increment of 10.4 °C/mW in its thermal resistance compared to that of FS1 (MHR=1, on partially etched substrate). This amounts to 650% increase in sensor’s thermal efficiency and decrease in conduction losses for this sensor on square membrane. Similar trend can be observed in previously published reports as well.

For example, a polysilicon heater with dimensions 150 µm × 3 µm × 0.25 µm and MHR = 1.33 had the sensitivity of 100 mV/Pa [67], whereas another polysilicon silicon heater having dimensions 80 µm × 2 µm × 0.3 µm and MHR = 3.12 has sensitivity of 1540 mV/Pa [76], despite the fact that the hot-film length in the latter case was almost half that of the former. Similarly, in another study [78], the thermal resistance of a titanium/platinum heater on silicon nitride membrane having MHR = 2 was 6.8 °C/mW, whereas our tungsten hot-film sensor FS3 (with MHR=2) on silicon oxide membrane achieved a thermal resistance 12 °C/mW, indicating a much better thermal isolation with silicon oxide membrane, which has lower thermal conductivity than silicon nitride. The reported thermal conductivity value of silicon oxide is 1.4 W/(m-K), whereas that for silicon nitride is 20 W/(m-K) [118], which in case of thin films may vary significantly depending upon the deposition parameters (e.g., thermal conductivity of a silicon nitride thin film is reported as 2.3 W/(m-K) [91], while that for silicon oxide thin film it is documented as 1.1 W/(m-K) [136]).

It is worth nothing, however, that thermal resistance (or conduction losses) of any hot-film flow sensor supported by a membrane depends upon both the membrane material (i.e., its thermal conductivity ‘k’) and membrane geometry (i.e., membrane shape, its MHR and thickness ‘t’).

For FS5 sensor with square membrane having MHR=3 (Figure 4), the decrease in the slopes with increasing voltages of I−V curve is more pronounced as compared with the FS3 sensor having MHR=2 (Figure 6). The thermal resistance of the FS5 hot-film sensor is 17.1 °C/mW (Figure 7). In comparison with the FS3 hot-films sensor having MHR=2, an increase in thermal resistance of 5.1 °C/mW has been achieved. This amounts to a further 42.5% increase in the sensor’s thermal efficiency (or decrease in conduction losses).

For the FS7 hot-film sensor (Figure 4) with square membrane having MHR=4, the decrease in the slopes with increasing voltages of I-V curves is even more prominent as compared with FS1, FS3 and FS5 hot-film sensors (Figure 6). The thermal resistance of the FS7 hot-film sensor is 19.4 °C/mW (Figure 7). The FS7 sensor with MHR=4 has achieved an increment of 2.3 °C/mW over the thermal resistance of FS5 sensor with MHR=3. This amounts to 12.8% increase in sensor’s thermal efficiency (or similar decrease in conduction losses).

It is worth noting that thermal resistance of the FS3 sensor (with MHR=2) increased by 650% compared to that of the FS1 (MHR=1, partially etched). This increment in thermal resistance for FS5 sensor (MHR=3) compared to that of the FS3 sensor (MHR=2) reduced to 42.5%. The improvement in sensor’s thermal efficiency (or thermal resistance) was further reduced to only 12.8% from FS5 sensor (MHR=3) to the FS7 sensor (MHR=4).

These experimental results thus point out that the gain in thermal efficiency (reduction in conduction losses or increase in thermal resistance) of the square membrane hot-film sensors for MHR > 3 is not much significant. However, at the same time, the increase in size of the sensor, which translates to increased price per sensor (as the price of CMOS sensor increases with the area occupied by it on CMOS processed wafer) and increase in sensor’s fragility increases drastically.

**Figure 9:**
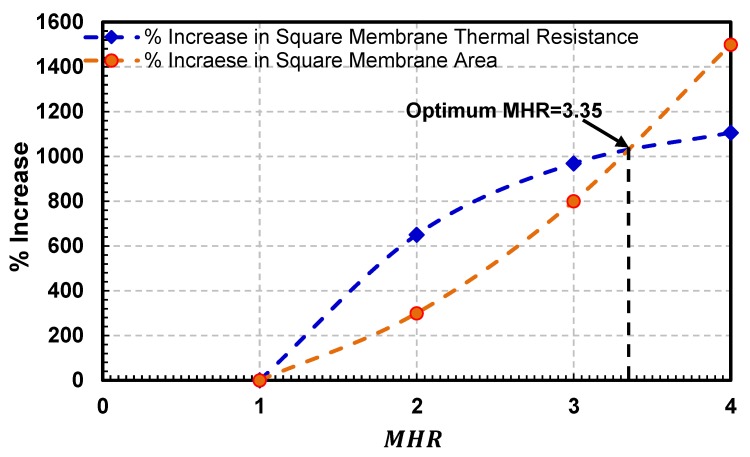
Plot showing the percentage increase in the square membrane area and the percentage increase in the thermal resistance with the increasing *MHR* for square membrane hot-film sensors FS1, FS3, FS5 and FS7. Up to MHR = 3.35, the percentage increase in sensors’ thermal resistance is more pronounced viz-a-viz the percentage increase in the membrane area. However, beyond MHR= 3.35, the change is opposite, thus using a MHR > 3.35 for square membranes is not very economical in terms of corresponding sharp increase in sensor size.

To identify the exact membrane to heater ratio (MHR) beyond which gain in sensor’s thermal efficiency is not economical in terms of excessively large chip area (or size), the percentage increase in square membrane sensors’ membrane area versus the percentage increase in their thermal efficiency (or thermal resistance) is plotted in Figure 9. Both the percentage increase in MHR and sensor area have been calculated with respect to the sensor FS1 having MHR=1.

It is evident from the figure that with increasing MHR, the percentage increase in the sensor’s thermal resistance is more pronounced as compared with the percentage increase in sensor (membrane) area till MHR value of 3.35. However, an increase in sensor (membrane) area associated with MHR values greater than 3.35 is not matched with similar increase in the thermal resistance of the sensor. Thus, increasing sensor MHR beyond this value for a gain in its thermal resistance (or reduction in conductive losses) is not very economical viz-a-viz a corresponding sharp increase in membrane area or sensors size.

**Figure 10:**
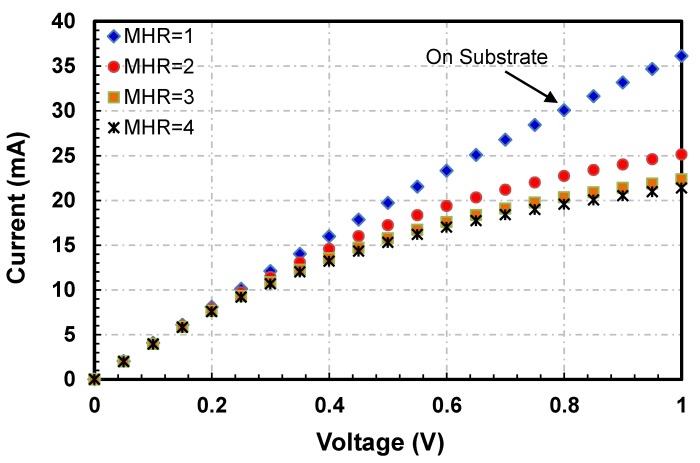
*I–-V* curves of sensors FS2, FS4, FS6 and FS8 having circular membranes: For *MHR* 1 (actually on substrate as membrane did not get etched), the *I–V* curve is almost linear indicating negligible change in sensor’s resistance. With increasing *MHR*, however, sensors’ Joule heating and resistances increase significantly.

**Figure 11:**
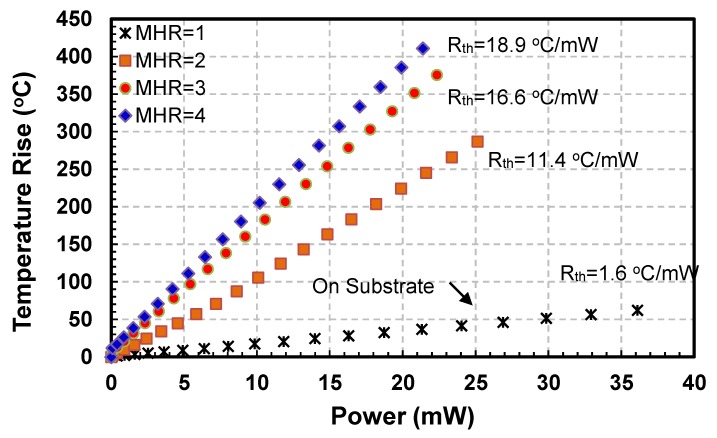
Power versus temperature rise curves of sensors FS2, FS4, FS6 and FS8 having circular membranes: For the FS2 sensor with *MHR* = 1 (on partially etched substrate), there is a negligible temperature rise in the sensor. There is a greater temperature rise between sensors with MHR=2 and 3, which reduces significantly for the sensor having *MHR* = 4.

The effect of MHR on the thermal efficiency of hot-film sensors having circular membranes is similar to that of the hot-film sensors having square membranes. The I−V curves for all four circular membrane hot-film sensors (i.e., FS2, FS4, FS6 and FS8 with MHR = 1, 2, 3 and 4, respectively) are shown in Figure 10 while P−T curves are presented in Figure 11.

For circular membrane hot-film sensor FS4 (Figure 3) having MHR=2, the I−V curve is nonlinear, with decreasing slopes with increasing voltages (Figure 10), like the behavior exhibited by the square membrane hot-film sensor FS3 as well (Figure 6). The thermal resistance of the FS4 hot-film sensor is 11.4 °C/mW (Figure 11). While comparing the temperature rise for this configuration with the former one (sensor on substrate), an increase of 9.8 °C/mW in sensor’s thermal resistance is recorded. This amounts to 512% increase in FS4 sensor’s thermal efficiency (and decrease in conduction losses) over that of FS2 (MHR=1) circular membrane sensor.

For circular membrane sensors having MHR=3, 4 (FS6 and FS8 sensor in Figure 4), the decrease in the slopes with increasing voltages of I−V curve is more pronounced as compared with the sensor having MHR=2 (Figure 10). The thermal resistance of the circular membrane hot-film sensors FS6 and FS8 is 16.6 °C/mW and 18.9 °C/mW, respectively (Figure 11). This amounts to 45.6% and 13.8% increase in thermal resistance of the circular membrane hot-film sensors from MHR=2 to MHR= 3 and from MHR=3 to MHR=4, respectively.

A qualitatively similar trend has been observed in the previously reported thermal flow sensors as well. A tungsten heater with a circular membrane having MHR=1.25 reported by Haneef et al. [83] had a sensitivity of 35 mV/Pa, whereas a tungsten heater with a circular membrane having MHR=3 reported by De Luca et al. [5] had a sensitivity of about 57.2 mV/Pa. It is worth noting, however, that the later had three thermopiles covering a large membrane area that provided an additional heat loss route through silicon based thermopiles’ to the membrane sides and substrate, due to which the hot-film sensor’s sensitivity was not that pronounced in spite of having a more efficient MHR (i.e., 3.0) compared to that of the former case (i.e., 1.25 only).

**Figure 12:**
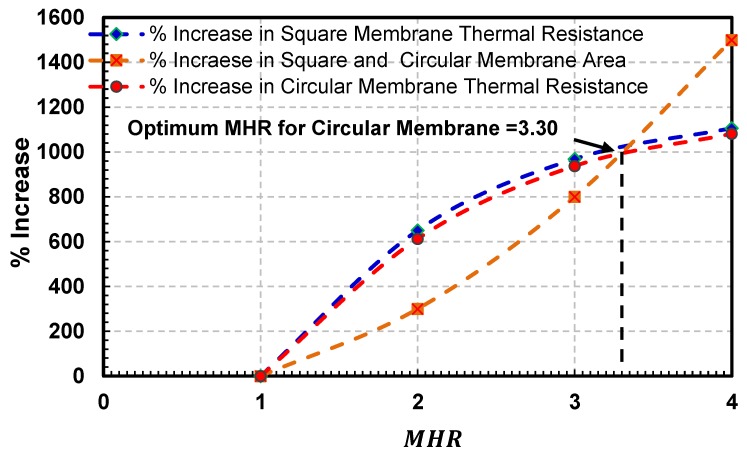
Plot showing the percentage increase in both square and circular membrane areas and increase in their thermal resistance with the increasing MHR. For circular membranes, the optimum MHR= 3.30, compared to the optimum MHR=3.35 for squaremembranes.

Similar to the square membranes case, the exact membrane to heater ratio (MHR) for circular membranes beyond which gain in sensors thermal efficiency is not very economical has been identified by plotting the percentage increase in sensors area against the percentage increase in the sensor thermal resistance (Figure 12).

It is evident from the figure that with increasing MHR, the percentage increase in the thermal resistance is more distinct in comparison with the percentage increase in the sensor (membrane) area till MHR=3.30. However, this increase is insignificant beyond MHR = 3.30, in comparison with a corresponding sharp increase in sensor (membrane) area. Thus increasing MHR beyond 3.30 is not viable.

**Figure 13:**
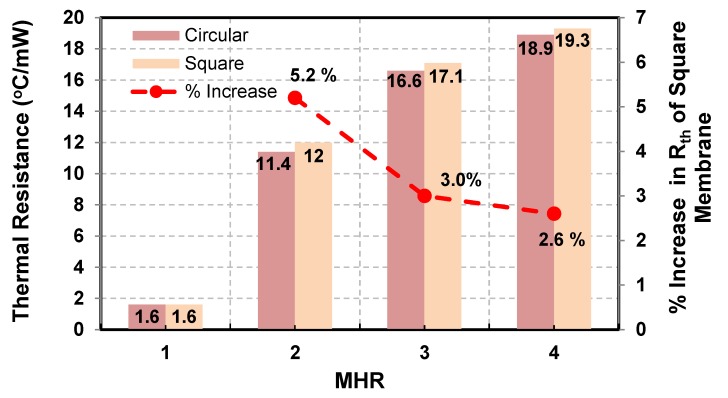
Thermal resistance (left y-axis) of square and circular membranes as a function of MHR compared on the bar graphs. Square membranes have relatively better thermal resistance. However with increasing *MHR*s, the % difference of thermal resistance between square and circular membranes (right y-axis) reduces significantly as shown by the line graph.

The effect of circular and square membrane shapes on thermal resistance (or conduction losses) of the hot-film sensors is given in Figure 13. In this figure, the sensors’ MHR is plotted against sensors’ thermal resistance (temperature rise per milli watts of supplied power) on left y-axis and % increase in the thermal efficiency of the square membrane as compared with that of the circular membrane on right y-axis. As shown in Figure 13, the thermal resistance of both type of membranes increases with increasing MHR. However, this increase is not very economical beyond a MHR= 3.35 for square membranes and MHR=3.30 for circular membranes. The performance of the square membrane is relatively better than the circular membrane. The incremental difference between the thermal efficiency of circular and square membrane hot-film sensors, however, decreases with an increase in MHR. Square membrane is 5.2% more efficient in terms of its thermal resistance than the circular membrane at MHR=2, which reduces to 3.0% and 2.6% at MHR=3 and MHR=4, respectively.

However, for higher membrane to heater ratios, it is more pragmatic to use circular membrane in hot-film flow sensors as it has a more uniform stress distribution at the edges with the substrate [6], thus achieving a better mechanical strength compared to that of the square membrane. It is pertinent to mention that for any specific mechanical loading, the maximum stresses generated on a square membrane are 64% higher than the circular membrane having the same material, thickness and diameter as of the side length of a square membrane [137].

## 5. Conclusions

Membrane heat conduction losses and their effects on SOI CMOS MEMS thermal (hot-film) flow sensor’s thermal resistance as a function of membrane shape and Membrane to Heater (hot-film length) Ratio (MHR=1, 2, 3, 4) have been investigated experimentally for the first time. For this purpose, electrical and thermal characterization of eight tungsten hot-film thermal flow sensors (four each having square and circular shapes) is carried out. The sensors were fabricated in a commercial 1 μm SOI CMOS foundry and then post-CMOS processed to create silicon oxide membranes under the hot-film sensors through a single DRIE back-etch step.

Experimental results demonstrate relatively lower conduction losses in square membranes-based hot-film MEMS flow sensors as compared with those having circular membranes. However, the conduction losses (or thermal resistance) difference between square and circular membranes decreases with increasing MHR. At MHR=2, the circular membrane hot-film sensor had 5.2% lower thermal resistance than that of the square membrane hot-film sensor, which reduced to 3.0% and 2.6% for square and circular membrane sensors having MHR=3 and MHR=4, respectively. Since square membranes experience much higher mechanical stresses than circular membranes (stresses on square membranes are 1.64 times of that on circular membranes), therefore, in spite of slightly better thermal resistance of square membranes, circular membranes may be the optimal choice for MEMS thermal hot-film flow sensors for achieving higher mechanical strength and robustness.

The thermal resistance of both square and circular membrane hot film sensors increases with increase in MHR. However, beyond a MHR=3.35 for square membranes and MHR=3.30 for circular membranes, experimental results suggest that the gain in sensor’s thermal resistance (reduction in conductive heat losses) is less significant as compared with the increase in the sensor (membrane) size, which adds to both the price and mechanical fragility of the sensor. It is therefore, neither cost effective nor mechanically preferable to have membrane to heater ratio (MHR) more than 3.35 and 3.30 for square and circular membranes-based SOI CMOS MEMS thermal hot-film flow sensors.

## Figures and Tables

**Table 1:** MEMS Thermal Flow Sensors Fabricated on Ceramic Substrates.

First Author, Year	[Ref]	Type	Sensor Material	Sensor Size (*l* × *w* × *t*, all in µm)	Substrate Material
Kuijk, 1995	[61]	TOF	Platinum	300 × 100 × NR	Glass
Tung, 2007	[99]	Hot-Film	MW CNT	360 × 90 × NR	Glass
Qu, 2008	[102]	Hot-Film	EG CNT	1 × 1 × 0.1	Glass
Zhu, 2015	[57]	Calorimetric	Platinum	NR × NR × 0.2	Silicon-in-Glass(SIG)
Makinwa, 2001	[15]	Calorimetric	Polysilicon	2000 × 200 × NR	Silicon/Ceramic for protection
Dominguez, 2008	[4]	Hot-Film	Platinum	NR × NR × 0.07	Silicon
*Makinwa, 2002	[17]	Calorimetric	Polysilicon	0.4 mm^2^ area	Silicon/Ceramic for protection
Matova, 2003	[47]	Calorimetric	P doped Silicon	NR × NR × NR	Silicon/Ceramic for protection
Kaltsas, 1999	[45]	Calorimetric	Polysilicon	NR × NR × NR	Porous Silicon
Kaltsas, 2002	[46]	Calorimetric	Polysilicon	NR × NR × NR	Porous Silicon
Stamatopoulos, 2008	[53]	Calorimetric	Polysilicon	NR × NR × NR	Porous Silicon
Sun, 2013	[112]	Hot-Film	Chromium/Platinum	1500 × 250 × 0.17	P-doped Silicon (Beam Material)
Wu, 2001	[95]	Hot-Film	Polysilicon	NR × NR × NR	SiN
Furjes, 2004	[48]	Calorimetric	Platinum	100 × 100 × 1	SiN
Dijkstra, 2008	[51]	Calorimetric	Platinum	NR × NR × 0.2	SiN
Wiegerink, 2009	[105]	Hot-Film	Aluminum	NR × NR × NR	SiN
Xiang, 2010	[55]	Calorimetric	Polysilicon	NR × NR × 1	Silicon
*Sun, 2007	[49]	Calorimetric	Polysilicon	600 × 60 × NR	Ceramic Al_2_O_3_
Shen, 2010	[56]	Calorimetric	Platinum	1000 × 1000 × 0.2	Ceramic (Exact ceramic name not reported)
Miau, 2015	[94]	Hot-Film	Platinum	200 × 260 × 0.1	Polyimide

Legend for Table 1, Table 2, Table 3, Table 4, Table 5 and Table 6. 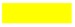 Hot-wire/Hot-film type thermal flow sensors; 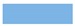 Calorimetric (Calori) type thermal flow sensors; 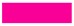 Time of flight (TOF) type thermal flow sensors; 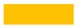 CMOS Sensors; 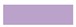 Sensors fabricated on Glass substrate; 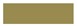 Sensors fabricated on Silicon substrate; 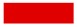 Sensors fabricated on Porous Silicon substrate; 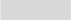 Sensors fabricated on Other Ceramic substrate; 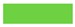 Sensors fabricated on Silicon Nitride substrate/Membrane; 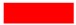 Sensors fabricated on Silicon Oxide substrate/Membrane; 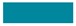 Sensors fabricated on Polyimide substrate/Membrane; 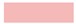 Sensors fabricated on Parylene substrate/Membrane; 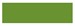 Sensors fabricated on Kapton substrate/Membrane; 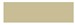 Sensors fabricated on Silicon Nitride/Silicon Oxide Membrane; SiN: Silicon Nitride; SiO: Silicon Oxide; *: CMOS Sensors; NR: Not Reported by the Author(s).

**Table 2:** MEMS Thermal Flow Sensors Fabricated on Polymer Substrates.

First Author, Year	[Ref]	Type	Sensor Material	Sensor Size *(l* × *w* × *t,* all in µm)	Substrate Material
Miau, 2006	[97]	Hot-Film	Platinum	260 × 200 × 0.1	Polyimide
Liu, 2007	[98]	Hot-Film	Platinum	2100 × 1500 × 500	Polyimide
Tan, 2007	[100]	Hot-Film	Gold//Chromium	4000 × 90 × 0.3	Polyimide
Buchner, 2008	[52]	Calorimetric	Titanium-Tungsten	NR × NR × NR	Polyimide
Liu, 2009	[106]	Hot-Film	Chromium/Nickel/Platinum	3000 × 300 × 0.23	Polyimide
Que, 2012	[111]	Hot-Film	Cr/Ni/Pt	NR × NR × NR	Polyimide
Li, 2015	[11]	Hot-Film	Gold	NR × NR × NR	Polyimide
Tang, 2016	[58]	Hot-Film	Platinum	NR × 5 × 0.2	Polyimide
Yu, 2016	[115]	Hot-Film	Chromium/Platinum	200 × 260 × 0.12	Polyimide
Yu, 2007	[101]	Hot-Film	Titanium/Platinum	160 × 80 × 0.1	Parylene C
Yu, 2008	[117]	Hot-Film	Titanium/Platinum	280 × 2 × 0.075	Parylene C
Chang, 2008	[103]	Hot-Film	Platinum	NR × NR × NR	Parylene C
Kuo, 2011	[110]	Hot-Film	Platinum	NR × NR × NR	Parylene C
Hasegawa, 2016	[114]	Hot-Film	Gold/Copper	NR × NR × 0.26	Parylene (Exact type NR)
Li, 2008	[104]	Hot-Film	Gold	NR × NR × 0.1	Kapton
Li, 2011	[9]	Hot-Film	Gold	NR × NR × 0.12	Kapton
Li, 2012	[10]	Hot-Film	Gold	NR × NR × 0.12	Kapton
Kaltsas, 2007	[50]	Hot-Film +Calorimetric	Platinum	NR × NR × 0.3	SU-8
Vilares, 2010	[54]	Calorimetric	Titanium/Platinum	2500 × 10 × 0.15	PMMA
Berthet, 2011	[62]	TOF	Titanium/Platinum	500 × 20 × 20	Pyrex

**Table 3:** MEMS Thermal Flow Sensors with Square Membranes.

First Author, Year	[Ref]	Type	Sensor Material	Sensor Size *(l* × *w* × *t,* all in µm)	Membrane Material	Membrane Size *(l* × *w* × *t,* all in µm)	MHR
Liu, 1994	[63]	Hot-Film	Polysilicon	100 × 2 × 0.45	SiN	200 × 200 × 1.2	2
Jiang, 1996	[65]	Hot-Film	Polysilicon	150 × 3 × 0.25	SiN	200 × 200 × 1.2	1.33
Huang, 1996	[64]	Hot-Film	Polysilicon	80 × 2 × NR	SiN	200 × 200 × 1.2	2.5
Jiang, 1997	[67]	Hot-Film	Polysilicon	150 × 3 × 0.25	SiN	200 × 200 × 1.2	1.33
Huang, 1999	[68]	Hot-Film	Polysilicon	(100,150,200) × (2,3,4) × 1	SiN	200 × 200 × 2	2
Liu, 1999	[69]	Hot-Film	Polysilicon	100 × 2 × 0.45	SiN	200 × 200 × 1.5	2
Hung, 2000	[70]	Hot-Film	Platinum	NR × NR × 0.18	SiN	600 × 600 × 0.2	-
Mailly, 2001	[71]	Hot-Film	Platinum	NR × NR × 0.3	SiN	650 × 650 × 0.5	-
Yoshino, 2001	[72]	Hot-Film	Platinum	200 × 23 × 0.1 300 × 32 × 0.1	SiN	400 × 400 × 1.0 500 × 500 × 1.0	2
Xu, 2002	[73]	Hot-Film	Polysilicon	150 × 2 × 0.5	SiN	210 × (45 -210) × 4.2	1.4
Xu, 2004	[75]	Hot-Film	Polysilicon	150 × 2 × 0.5	SiN	210 × (75 -210) × 4.2	1.4
Xu, 2005	[123]	Hot-Film	Polysilicon	150 × 7 × 0.5	SiN	210 × 210 × 1.5	1.4
Xu, 2005	[124]	Hot-Film	Polysilicon	180 - 210 × NR × 0.5	SiN	210 × 45 - 210 × 4.2	1 – 1.16
Soundrarajan, 2005	[76]	Hot-Film	Polysilicon	80 × 2 × 3	SiN	NR × NR × 0.3	-
Kim, 2006	[77]	Hot-Film	Gold	600 × 50 × 0.45	SiN	1000 × 1000 × 1.3	1.66
Liang, 2008	[78]	Hot-Film	Titanium/Platinum	100 × 2 × 0.2	SiN	200 × 200 × 1.5	2
Sabate, 2004	[25]	Calorimetric	Nickel	NR × 40 × 0.15	SiN	750 × 750 × 0.3	-
Buchner, 2006	[26]	Calorimetric	Polysilicon	NR × NR × 0.3	SiN	1000 × 1000 × NR	-
Adamec, 2010	[39]	Calorimetric	Nickel	NR × NR × NR	SiN	NR × NR ×NR	-
Sosna, 2010	[59]	TOF	NR	1000 × 10 × 0.3	SiN	1000 × 1000 × 0.6,0.3 600 × 800 × 0.6	1
Sosna, 2011	[60]	TOF	NR	1000 × 10 × 0.3	SiN	1000 × 1000 × 0.6,0.3	1
Laconte, 2004	[24]	Calorimetric	Polysilicon	240 × 240 × 0.34 (Active area)	SiN/SiO	440 × 440 × 1 640 × 640 × 1 840 × 840 × 1	1.83
Yu, 2008	[27]	Calorimetric	Platanium	NR × NR × NR	SiN/SiO	1800 × 1800 × NR	-
Cubckcu, 2010	[3]	Calorimetric	Germanium	NR × NR × NR	SiN/SiO	1000 × 1000 × 1.4	-
Hsiai, 2004	[8]	Hot-Film	Polysilicon	80 × 2 × 0.5	SiN/SiO	100 × 100 × 1.5	1.25
*Piotto, 2012	[28]	Calorimetric	Polysilicon	NR × NR × NR	SiO	NR × NR × NR	-
*Haneef, 2007	[125]	Hot-Film	Aluminum	130 × 3 × 0.72	SiO	500 × 500 × NR	3.84
*Haneef, 2008	[126]	Hot-Film	Aluminum	130 × 3 × 0.72 18.5 × 1.1 × 0.72	SiO	500 × 500 × NR 266 × 266 × NR	3.8414.3
Kalvesten, 1996	[66]	Hot-Film	Polysilicon	300 × 60 × 30	Polysilicon	1500 × 1500 × 30	5
*Xu, 2003	[74]	Hot-Film	Polysilicon	200 × NR × 0.32	Parylene N	250 × 100 × 1.5	1.25

**Table 4:** MEMS Thermal Flow Sensors with Circular Membranes.

First Author, Year	[Ref]	Type	Sensor Material	Sensor Size (*l* × *w* × *t,* all in µm)	Membrane Material	Membrane Size (*Dia* × *t,* all in µm)	MHR
Breuer, 1999, 2000	[79,80]	Hot-Film	Platinum	100 × 5 × 0.1	SiN	[210 × 0.15]	2.1
Qu, 2016	[22]	Hot-Film	Platinum	140 × NR × NR	SiN	NR × NR × 1.5	-
Cain, 2000	[81]	Hot-Film	Platinum	200 × 4 × 0.15	SiN	[200 × 0.15]	1
Cubckcu, 2010	[3]	Calorimetric	Germanium	NR × NR × NR	SiN/SiO	[1000 × 1.4]	-
Reyes-Romero, 2013	[30]	Calorimetric	Chromium	NR × NR × NR	SiN/SiO	[1000 × 1.4]	-
Reyes-Romero, 2013	[31]	Calorimetric	Chromium	NR × NR × NR	SiN/SiO	[1000 × 1.4]	-
*Haneef, 2014	[83]	Hot Film	Tungsten	200 × 2 × 0.3	SiO (with SiN passivation)	[250 × NR]	1.25
*De Luca, 2013	[5]	Calorimetric+ Hot-Film	Tungsten	400 × 2 × NR	SiO	[1200 × NR]	3
*De Luca, 2015	[6]	Calorimetric	Tungsten	400 × 2 × NR	SiO	[1200 × NR]	3
Fan, 2004	[82]	Hot-Film	Gold	[NR × 0.2]	Parylene C	[400 × 12]	-

**Table 5:** MEMS Thermal Flow Sensors with Rectangular Membranes.

First Author, Year	[Ref]	Type	Sensor Material	Sensor Size (*l* × *w* × *t,* All in µm)	Membrane material	Membrane Size (*l* × *w* × *t,* all in µm)	MHR
Yoshino, 2003	[85]	Hot-Film	Platinum	250 × 30 × 0.1	SiN	350 × 200 × 1.0	1.4
Ma, 2009	[89]	Hot-Film	Platinum	4400 ID ×300 × 0.1	SiN	NR × NR × 1	-
Saremi, 2014	[91]	Hot-Film	Platinum	2700 × 13 × 0.3	SiN	3000 × 1000 × 450	1.11
Shi, 2006	[86]	Hot-Film	Polysilicon	200 × 4 × 0.45	SiN	250 × 200 × 1.5	1.25
Ernst, 2002	[127]	Calorimetric	Germanium	574 × 6 × 0.2	SiN	900 × 700 × 1.4	1.56
Hedrich, 2010	[33]	Calorimetric	Polysilicon	NR × NR × 0.3	SiN	300 × 600 × 0.15	-
Dalola, 2012	[34]	Hot-Film and Calorimetric	Germanium	600 × 35 × 0.26	SiN/SiO	1000 × 500 × 1.6	1.66
Talic, 2015	[42]	Calorimetric +Hot-Film	Chromium	NR × NR × 0.13	SiN/SiO	1000 × 500 × 1.57	-
Bruschi, 2005	[32]	Calorimetric	Polysilicon	NR × NR × NR	SiO	45 × 60 × NR	-
Liu, 2013	[13]	Hot-Film	Titanium/Platinum	NR × NR × 0.11	SiO	NR × NR × NR	-
*Wang, 1999	[84]	Hot-Film	Polysilicon	NR × NR × 0.32	Parylene N	NR × NR × 3.5 NR × NR × 0.7	-
Wu, 2016	[23]	Hot-Film	Platinum	NR × 10 × 0.5	Parylene C	NR × NR ×NR	-
Shibata, 2014	[92]	Hot-Film	Gold/copper	NR × NR × 0.26	Polyimide	NR × NR × 5	-
Imaeda, 2015	[93]	Hot-Film	Gold/Copper	NR × NR × 0.26	Polyimide	1600 × 1700 × 5	-
Hepp, 2011	[40]	Calorimetric	Platinum	NR × NR × NR	Polyimide	NR × NR × 6	-
Strum, 2013	[41]	Calorimetric	Tungsten -Titanium	NR × NR × NR	Polyimide	200 - 600 × 800 × 9.6	-
Meng, 2008	[36]	TOF Calorimetric Hot-Film	Platinum	200 × 25 × 0.1	Parylene C	NR × NR × 6	-
Sturm, 2010	[38]	Calorimetric	Titanium/ Tungsten	NR × NR × NR	SiN/Polyimide	NR × NR ×NR	-
Etxebarria, 2016	[44]	Calorimetric	Nickel	NR × NR × 0.1	Polymer (Exact name NR)	NR × NR ×NR	-
Nguyen, 1997	[35]	Calorimetric	Polysilicon	NR × NR × NR	Silicon	NR × NR ×NR	-

**Table 6:** Geometric Parameters of the Fabricated SOI CMOS MEMS Thermal Hot-Film Sensors.

SensorNomenclature	Membrane Shape	Membrane Size (Side Length/Diameter) µm	Heater (Hot-Film) Size (l × w × t) µm	Membrane to Heater Ratio (MHR)
FS1	Square	80	All heaters/hot-films have the same size (80 × 2 × 0.3)	1
FS2	Circular			
FS3	Square	160		2
FS4	Circular			
FS5	Square	240		3
FS6	Circular			
FS7	Square	320		4
FS8	Circular			
